# Personals

**Published:** 1917-12

**Authors:** 


### PERSONAL

Announcement of removal, travel, and other matters of interest are requested. Please report errors in the listing of any physician in the State and other directories, that we may co-operate with the State Society in securing a correct list.

Dr. Frank B. Storer of Holley is spending the winter in California.

Drs. E. P. H. Griswold and E. O. Bingham of Niagara Falls have been appointed to the municipal civil service commission, vice Drs. F. N. C. Jerauld and R. S. Barry, now in military service.

Dr. Wm. B. Johnston of Ellicottville spent some time hunting in the Adirondacks late in October and early in November.

Dr. Ira W. Livermore of Gowanda was home from Camp Bartlett, Westfield, Mass., early in November.

Dr. Lee Masten Francis of Buffalo was elected Secretary of the American Academy of Ophthalmology and Oto-Laryngology.

In our personal notice in the November issue, of the removal of the office of Dr. John D. Flagg of Buffalo to 473

Virginia St., it was incorrectly stated that his practice was limited to Laryngology, Ophthalmology was meant.

Dr. Edgar R. McGuire of Buffalo announces the removal of his office and residence to 622 Delaware Ave.

Dr. Joseph S. Lewis of Buffalo was injured in a collision with a trolley November 14. He sustained a fracture of the clavicle and was unconscious for a short time. He was taken to the Homoeopathic Hospital. At last account he was progressing favorably.

Dr. Stephen Y. Howell is spending a short time at the Mayo Clinic, Rochester, Minn.

The Supervisors of Cattaraugus Co. at their meeting of November 14, increased the salary of the jail physician from $125 to $300. Dr. M. L. Hillsman is the present incumbent.

